# FolVps9, a Guanine Nucleotide Exchange Factor for FolVps21, Is Essential for Fungal Development and Pathogenicity in *Fusarium oxysporum* f. sp. *lycopersici*

**DOI:** 10.3389/fmicb.2019.02658

**Published:** 2019-11-14

**Authors:** Bing Li, Hui-Ying Mao, Zhao-Yang Zhang, Xi-Jun Chen, Shou-Qiang Ouyang

**Affiliations:** ^1^College of Horticulture and Plant Protection, Yangzhou University, Yangzhou, China; ^2^Joint International Research Laboratory of Agriculture and Agri-Product Safety of the Ministry of Education of China, Yangzhou University, Yangzhou, China; ^3^Key Laboratory of Plant Functional Genomics of the Ministry of Education, Yangzhou University, Yangzhou, China

**Keywords:** *Fusarium oxysporum*, FolVps9, guanine nucleotide exchange factor (GEF), plant infection, endocytosis, autophagy

## Abstract

The soil-borne, asexual fungus *Fusarium oxysporum* f.sp. *lycopersici* (*Fol*) is the causal agent of tomato wilt disease. Autophagy plays a crucial role in the development and virulence of *Fol*. The *Fol* endosomal system is highly dynamic and has been shown to be associated with conidiogenesis and pathogenicity. Rab GTPases and the regulators are highly conserved in regulating autophagy and endocytosis in most eukaryotes. Identification and characterization of additional Rab regulators in fungal pathogens should facilitate the understanding of the autophagy and endocytosis in different filamentous fungi. Here, we have identified and characterized the yeast *VPS9* homolog *FolVPS9* in *Fol*. Targeted gene deletion showed that *FolVPS9* is important for growth, conidiation and virulence in *Fol*. Cytological examination revealed that FolVps9 co-localized with FolVps21 (a marker of early endosome) and played a critical role in endocytosis and autophagosome degradation. Pull-down assays showed that FolVps9 interacted with FolVps21, which was also important for development and plant infection in *Fol*. Yeast two-hybrid, bimolecular fluorescence complementation and co-immunoprecipitation assays revealed that FolVps9 specifically interacts with the GDP-bound form of FolVps21. Furthermore, a constitutively active form of FolVps21 (Q72L) was able to rescue defects of Δ*Folvps9* and Δ*Folvps21* mutants. In summary, our study provides solid evidence that FolVps9 acts as a FolVps21 guanine nucleotide exchange factor (GEFs) to modulate endocytosis and autophagy, thereby controlling vegetative growth, asexual development and pathogenicity in *Fol.*

## Introduction

Endocytosis is a conserved transport process in which macromolecules, lipids, or membrane proteins are transported to endosomal compartments. Recently, a series of studies have shown that endocytosis plays an important role in cell polarity, signal transduction and plant infection in phytopathogens (Wedlich-Soldner et al., 2000; Ramanujam et al., 2013; Li L. et al., 2017; Li et al., 2019b). During endocytosis, cargo is packaged by an area of plasma membrane, which then buds off inside the cell to form transport vesicles (Balderhaar and Ungermann, 2013; Li X. et al., 2017). These transport vesicles transporting the cargo from inside the cell membrane to the early endosome undergo a sorting process, in which they are either trafficked back to the plasma membrane or sent to the vacuole (Seaman, 2008; Li X. et al., 2017; Zhu et al., 2018).

Previous studies have shown that Rab GTPases regulate endocytic vesicle trafficking by targeting these vesicles to appropriate cellular compartments (Balderhaar and Ungermann, 2013; Ramanujam et al., 2013; Zheng et al., 2015). For example, the early stage of the endocytic pathway is regulated by Rab5, while Rab7 controls the late stage (Balderhaar and Ungermann, 2013; Solinger and Spang, 2013). Rab proteins exist in both the active GTP-bound and inactive GDP-bound states, which are regulated by guanine nucleotide exchange factor (GEFs) and GTPase-activating proteins (GAPs) (Zerial and McBride, 2001; Markgraf et al., 2007; Zheng et al., 2018a). GEFs facilitate formation of the GTP-bound form of the Rab protein; in this state, the Rab protein can bind multiple tethering effectors to promote vesicle trafficking (Ishida et al., 2016). Conversely, GAPs catalyze hydrolysis of GTP to GDP on the Rab protein, thereby converting it to the GDP-bound conformation (Cai et al., 2007; Ishida et al., 2016). Based on sequence similarity and their cognate GTP-binding protein, GEFs have been divided into different families (Cherfils and Chardin, 1999; Hutagalung and Novick, 2011; Ishida et al., 2016). To date, more than 40 putative Rab GEFs have been identified in humans, and they all possess at least three conserved domains, including the Vps9, DENN, and Sec2 motifs (Ishida et al., 2016).

In *Saccharomyces cerevisiae*, there are at least three Vps9 domain proteins (Vps9p, Muk1p, and Vrl1p) that function as Rab5 GEFs (Bean et al., 2015). Vps9 was the first GEF identified, and it has been shown to bind retromer (an endosome-localized complex involved in protein retrograde) to facilitate recruitment of PI3P (phosphatidyl-inositol-3-phosphate) to endosomes (Bean et al., 2015). Subsequent studies have shown that Vps9p is a vacuolar sorting protein that contains a Vps9 domain, which is involved in vesicle-mediated vacuolar protein trafficking (Hama et al., 1999). Previous studies have shown that Vps9p, Muk1p and Vrl1p have redundant, yet distinct functions (Bean et al., 2015). Deletion of *MUK1* does not result in a visible phenotype in yeast. However, the *VPS99 MUK1* double mutant displays a more severe phenotype than the *VPS9* single mutant (Paulsel et al., 2013). In addition, overexpression of *MUK1* or *VRL1* fully restores the temperature-sensitive growth phenotype of Δ*vps9*, but cannot replace the role of Vps9 in late endosomal sorting of carboxypeptidase Y (CPY; Paulsel et al., 2013; Bean et al., 2015).

Cross-talk between autophagy and endocytosis exists and there is evidence demonstrating that the same proteins play important roles in both processes (Lipatova et al., 2012; Sarkar, 2013). However, the endocytosis pathway is mainly responsible for absorption and utilization of nutrients from outside the cell, while the autophagy pathway is mainly responsible for the degradation of endogenous waste components from the cytoplasm. During autophagy, cellular components are packaged by coated vesicles to form autophagosomes, which then fuse with the vacuole to facilitate degradation (Voigt and Poggeler, 2013). In addition to autophagy-related proteins (Atgs), other proteins that regulate vesicle trafficking, including Rab and its GEFs, are essential for autophagy (Gao et al., 2013; Bean et al., 2015; Li et al., 2015; Liu et al., 2015; Zheng et al., 2015). In *S. cerevisiae*, the Rab5p/Vps21p module controls formation of autophagosomes and Rab7 regulates fusion of autophagosomes with the vacuole (Chen et al., 2014; Zhou et al., 2017).

*Fusarium oxysporum* f.sp. *lycppersici* (*Fol*) is a soil-borne vascular fungal pathogen, which causes wilting disease or root rot on more than 100 different plants, including tomato, cotton, banana, melons and peppers (Michielse and Rep, 2009; Ouyang et al., 2014; Gonzalez-Cendales et al., 2016; Li et al., 2019b). Based on host specificity, *F. oxysporum* has been grouped into *forma speciales* (f.sp.) (van Dam et al., 2016). Although a connection in *Fol* is currently unknown, previous studies have shown that autophagy and endocytosis play essential roles in development and pathogenicity in other phytopathogens (Dou et al., 2011; Liu et al., 2015; Zhang et al., 2016). For example, the Rab GTPase regulates autophagy and endocytosis by controlling membrane trafficking in the plant pathogens *Magnaporthe oryzae* and *Fusarium graminearum* (Liu et al., 2015; Zheng et al., 2015). Of particular note, the Rab7 GTPase and its GEF Mon1 are known to be required for fungal vacuolar fusion, autophagy and plant infection in *M. oryzae* and *F. graminearum* (Gao et al., 2013; Li et al., 2015).

Identification and analysis of additional Rab regulators in fungal pathogens will be helpful to further understand vesicle trafficking, pathogenesis and how Rab is controlled in different filamentous fungi. In this study, we have identified and characterized FolVps9 from the tomato pathogen *Fol* and showed that the protein was required for fungal development, endocytosis, autophagy and plant pathogenicity. Based on genetic evidence and cytological examination, our results support a model in which FolVps9 is a GEF for FolVps21. In particular, we showed that constitutively activated *FolVps21* was able to rescue the defects of the Δ*Folvps9* mutant. In addition, FolVps21 is also required for normal fungal development and plant infection and the FolVps9-FolVps21 interaction plays an important role in endocytosis and autophagy of *Fol*.

## Materials and Methods

### Strains and Culture Conditions

Wild type *F. oxysporum* f.sp. *lycopersici* (*Fol*) strain 4287 and mutant strains used in this study are listed in Supplementary Table S1. Vegetative growth rate was determined using potato dextrose agar (PDA) and complete medium (CM) at 28°C for 5 days (Li et al., 2019b). Conidiation was assayed as previously reported (Li et al., 2019b). The conidial morphology of wild type and mutants was examined using a Zeiss LSM 710 confocal microscope with a 63/1.2 NA C-Apochromat oil immersion objective (Zeiss, Oberkochen, Germany). Liquid CM was used to grow cultures for extraction of genomic DNA and total RNA.

### Fungal Transformation and Gene Deletion

*F. oxysporum* protoplast preparation and fungal transformation were performed as previously described (Li et al., 2019a). The split-marker PCR approach was used to generate targeted gene replacement vectors (Catlett et al., 2003; Fang et al., 2018; Yang et al., 2018). Primers used for amplifying flanking sequences for each gene are listed in Supplementary Table S2. These primers were used to amply fragments for transformation using PCR, after which the PCR products were purified and transformed into protoplasts of wild type strain Fol4287. Hygromycin B (Solarbio, Beijing, China) was added to agar medium at a final concentration of 300 μg ml^–1^ for transformant selection. The putative targeted gene deletion mutants were screened by PCR assays with two pairs of primers in Supplementary Table S2 and further confirmed by Southern blots.

### Vector Construction

Unless noted otherwise, all fluorescent protein fusion vectors were constructed with the entire protein coding sequence under the control of RP27 constitutive promoter. Fragments were amplified and transformed with *Xho*I-digested pYF11 into *S. cerevisiae* strain XK*1-25* and final vector assembled using the yeast gap repair approach. For the FolVps21 domain deletion and the S27N, Q72L point mutation vectors, primers were designed to use in Splicing Overlap Extension (SOE)-PCR to create the mutations, amplifying sequences from the RFP-*FolVPS21* fusion vector. The PCR products were cloned into the pYF11 plasmid using yeast gap repair and verified by sequencing analysis. The primers used in this section are listed in Supplementary Table S2.

### Virulence Assays

For tomato root inoculation assays, 12 days old seedlings (Moneymaker, susceptible *i-*2) were carefully uprooted and washed in tap water to remove soil particles. The tomato seedling roots were inoculated using a conidial suspension (5 × 10^6^ conidia ml^–1^) or mock (water) for 30 min. The treated seedlings were then planted in vermiculite and maintained in a growth chamber. Disease symptoms were examined and photographed 3 weeks after inoculation. Pathogenicity experiments were performed three times with similar results. Fungal recovery assays were performed as previously described (Fradin et al., 2009; Li et al., 2019b). Root vascular bundles were observed using a Hitachi TM-1000 tabletop microscope.

### Purification of FolVps9 Complexes by Affinity Purification

To identify FolVps9-interacting proteins, a strain expressing FolVps9-3x*FLAG* was constructed. Expression of FLAG-tagged FolVps9 in the strain was confirmed by western blot analysis using anti-FLAG antibody. A positive expressing strain was cultured in 150 ml of CM liquid medium with shaking at 28°C for 24 h. Mycelia were harvested, ground into powder and total proteins isolated using extraction buffer [50 mM Tris-HCl (pH 7.5), 100 mM NaCl, 5 mM EDTA, 1% Triton X-100, 1 mM protease inhibitor cocktail (Sigma-Aldrich), and 1 mM phenylmethylsulfonyl fluoride (PMSF)]. The resulting mixture was centrifuged at 4°C for 30 min at 20,000 × *g*. The supernatant was incubated with anti-FLAG M2 agarose beads (Sigma-Aldrich) at 4°C for 6 h, and washed three times with wash buffer [50 mM Tris-HCl (pH 7.5), 150 mM NaCl, 0.5 mM EDTA]. The proteins were eluted with 200 mM glycine (pH 2.5) and then digested with trypsin and analyzed by Liquid Chromatography-Mass Spectrometry/Mass Spectrometry (LC-MS/MS) as described (Li B. et al., 2017). The resulting MS data were analyzed and searched against the non-redundant *F. oxysporum* f.sp. *lycopersici* protein database at NCBI.

### Yeast Two-Hybrid (Y2H), Co-immunoprecipitation (Co-IP) and Bimolecular Fluorescence Complementation (BiFC) Assays

The yeast two-hybrid assay was carried out using the Matchmaker Gal4 two-hybrid system 3 (Clontech, United States). Wild type FolVps21-AD, FolVps21^S27N^-AD and FolVps21^Q72L^-AD were cloned into pGADT7 (prey construct), while wild type FolVps9-BD, FolVps9^Δ^ ^Vps9^-BD and FolVps9^Δ^ ^GUE^-BD were cloned into pGBKT7 (bait construct). The pairs of prey and bait constructs were co-transformed into the yeast strain AH109 as previously described (Li B. et al., 2017). pGADT7-T and pGBKT7-53 were used as positive controls and pGADT7-T and pGBKT7-lam were negative controls. The Trp^+^ and Leu^+^ yeast transformants were isolated and assayed for growth on SD dropout medium lacking tryptophan, leucine, histidine and adenine (SD-Trp-Leu-His-Ade).

For co-immunoprecipitation assays, the *FolVPS9* ORF was cloned into pHZ126 to generate the 3 × *FLAG* fusion construct and the FolVps21^S27N^ ORF was cloned into pYF11 to generate the GFP fusion construct using the yeast gap repair approach. The resulting constructs were verified by sequencing analysis and subsequently co-transformed into protoplasts of Fol4287. Hygromycin B and G418 (Solarbio, Beijing, China) were added at a final concentration of 300 μg ml^–1^ for transformant selection. The transformants were confirmed by western blot assays using anti-GFP or anti-FLAG antibody (Abcam, Cambridge, MA, United States). Total proteins were extracted and incubated with GFP beads (Sigma-Aldrich) as previously described (Li L. et al., 2017). Proteins eluted from beads were analyzed by western blot with anti-GFP (GFP-Tag Mouse mAb, Abmart, China) or anti-FLAG antibody (FLAG-Tag Mouse mAb, Abmart, China), respectively. Results were visualized with the ECL detection system (Amersham Biosciences).

For BiFC assays, the FolVps9-YFP^C^ and FolVps21^S27N^ -YFP^N^ fusion constructs were made by cloning fragments containing the FolVps9 and FolVps21^S27N^ ORFs along with their native promoter regions (1500 bp upstream from the start codon) into pHZ65 or pHZ68, respectively. The resulting constructs were verified by sequencing analysis and co-transformed into protoplasts of Fol4287. Hygromycin B and G418 (Solarbio, Beijing, China) were added at a final concentration of 300 μg ml^–1^ for transformant selection. The YFP signal was observed using a fluoresence microscope. All primers used in this section were listed in Supplementary Table S2.

### Staining and Cellular Localization Assays

To examine endocytosis, hyphae of the indicated strains were cultured in liquid CM medium at 28°C for 16 h and stained with FM4-64 following procedures previously described (Fischer-Parton et al., 2000). Photographs were taken using a confocal laser scanning microscope (Zeiss, Oberkochen, Germany). For vacuole staining, CMAC (7-amino-4-chloromethylcoumarin; Sigma-Aldrich; stock solution in 10 mM dimethyl sulfoxide) was used to stain hyphae cultured as described above (Zhang et al., 2016).

### Autophagy Assays

For GFP-FolAtg8 and cleaved GFP assays, the GFP-*FolATG8* vector was transformed into the Fol4287 wild type strain and various mutants using the polyethylene glycol (PEG)-mediated transformation as previously described. Transformants expressing GFP-FolAtg8 were grown in CM medium for 24 h and then shifted to nitrogen starvation (MM-N) for 8 h to induce non-selective autophagy. Hyphae induced for 8 h were analyzed for GFP fluorescence using a fluorescent microscope. GFP-FolAtg8 proteolysis was assessed by western blot using GFP antibody (GFP-Tag Mouse mAb, Abmart, China). The amount of free GFP and GFP-FolAtg8 was calculated using grayscale analysis (Image-pro plus; Media Cybernetics, Shanghai, China) after western blotting.

### Quantitative Real-Time PCR (qRT-PCR) Analysis

Total RNA was extracted from tomato roots using the TRIzol LS reagent (Invitrogen) according to the manufacturer’s recommendations, and subsequently further purified using RNeasy Mini spin columns (Qiagen). Synthesis of first-strand cDNA was performed as previously described (Li et al., 2019a). The relative quantification of each transcript was calculated using the 2^–ΔΔ^ ^CT^ method (Livak and Schmittgen, 2001). All qRT-PCR assays were conducted in triplicate for each sample and the experiment was repeated three times. All primers used in this section are listed in Supplementary Table S2.

### Statistical Analysis

Each result was presented as the mean ± standard deviation (SD) of at least three replicated measurements. Significant differences between treatments were statistically evaluated by SD and one-way analysis of variance (ANOVA) using SPSS 2.0. The data for two different treatments were compared statistically by ANOVA, followed by the *F*-test if the ANOVA result was significant at *p* < 0.01.

### Accession Number

Gene sequences are available using the following accession number^1^ : *FolVPS9* (FOXG_00470), *FolVPS21* (FOXG_09392).

## Results

### Identification and Deletion of *FolVPS9* in *F. oxysporum* f. sp. *lycopersici*

Using the *S. cerevisiae* Vps9p protein sequence as the reference to blast the *F. oxysporum* f.sp. *lycopersici* genome database, we identified FOXG_00470 as a homolog of *S. cerevisiae* Vps9p that we named *FolVPS9*. *FolVPS9* is predicted to contain 2548 base pairs with two introns, and encode a polypeptide of 783 amino acids (aa). Domain analysis revealed that FolVps9 possesses Vps9 (480-595 aa) and ubiquitin-binding domains (CUE, 741–782 aa)^2^. To explore the physiological function of Vps9 in *F. oxysporum* f.sp. *lycopersici*, we used a gene replacement construct to mutate *FolVPS9* (Supplementary Figure S1A). The Δ*Folvps9* mutant was confirmed by Southern blot analysis (Supplementary Figure S1B). The mutation was also complemented by re-introducing the full length *FolVPS9* gene (Δ*Folvps9/FolVPS9* strain) and showing that this construct restored wild type functions.

### FolVps9 Is Important for Vegetative Growth and Asexual Reproduction

**FIGURE 1:**
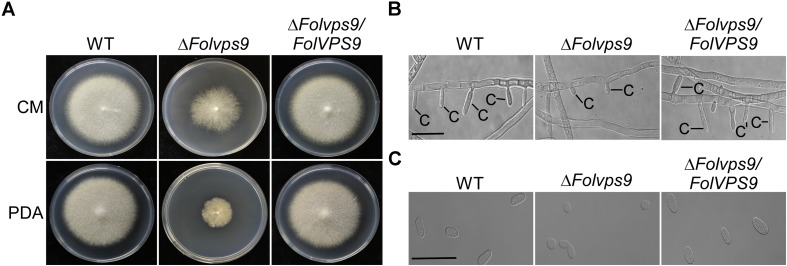
FolVps9 is involved in vegetative hyphal growth and asexual development. **(A)** Colony morphology. Wild type, Δ*Folvps9* mutant and the complemented strains were grown on CM and PDA media at 28°C for 5 days in the dark. **(B)** Conidiophore development. The indicated strains were cultured in CMC liquid medium for 5 days and photographed under differential interference contrast (DIC) microscopy. Bar = 10 μm. C, conidium. **(C)** Microconidia morphology. Wild type, Δ*Folvps9* mutant and the complemented strains were cultured in CMC liquid medium for 5 days and observed by DIC microscopy. Bar = 5 μm.

To explore the influence of *FolVPS9* on vegetative growth, we first checked the growth and colony morphology of the Δ*Folvps9* mutant on CM and PDA agar plates. After 5 days of incubation in the dark at 28°C, the Δ*Folvps9* mutant exhibited significantly reduced radial growth on CM and PDA media (Figure 1A). The colony diameter was reduced by 53.9 and 44.1% on CM and PDA plates, respectively (Table 1). This result indicates that FolVps9 is important for normal vegetative growth.

**TABLE 1:** Characterization of growth and conidiation in *F. oxysporum f. sp. lycopersici* strains.

	**Colony diameter (cm)^a^**		**Microconidia**	**Macroconidia**	**Microconidia length**
				
**Strain**	**CM**	**PDA**	**(×10^8^/ml)^b^**	**(×10^3^/ml)^c^**	**(μm)^d^**
WT	6.3 ± 0.2	5.9 ± 0.1	3.1 ± 0.2	0.6 ± 0.1	9.5 ± 2.1
Δ*Folvps9*	3.4 ± 0.3^∗^	2.6 ± 0.2^∗^	1.2 ± 0.1^∗^	0.3 ± 0.1^∗^	4.8 ± 1.8^∗^
Δ*Folvps21*	2.7 ± 0.2^∗^	2.3 ± 0.1^∗^	1.5 ± 0.2^∗^	0.5 ± 0.2	5.1 ± 2.3^∗^
Δ*Folvps9/FolVPS9*^DVPS9^	NA	2.9 ± 0.1^∗^	1.0 ± 0.3^∗^	0.2 ± 0.1^∗^	NA
Δ*Folvps9/FolVPS9*^DCUE^	NA	6.0 ± 0.2	3.1 ± 0.2	0.5 ± 0.2	NA
Δ*Folvps9/FolVPS21*^S27N^	NA	2.7 ± 0.1^∗^	1.2 ± 0.2^∗^	0.2 ± 0.1^∗^	4.5 ± 2.1^∗^
Δ*Folvps9/FolVPS21*^Q72L^	NA	2.6 ± 0.1^∗^	3.0 ± 0.3^∗^	0.3 ± 0.2^∗^	9.5 ± 2.1
Δ*Folvps9/FolVPS21*	NA	2.7 ± 0.3^∗^	1.3 ± 0.2^∗^	0.3 ± 0.1^∗^	4.7 ± 1.9^∗^
Δ*Folvps21/FolVPS21*^S27N^	NA	2.2 ± 0.2^∗^	1.6 ± 0.1^∗^	0.2 ± 0.1^∗^	4.9 ± 2.5^∗^
Δ*Folvps21/FolVPS21*^Q72L^	NA	6.1 ± 0.2	3.1 ± 0.2	0.5 ± 0.2	9.3 ± 2.0
Δ*Folvps9/FolVPS9*	6.1 ± 0.2	6.0 ± 0.3	3.2 ± 0.1	0.6 ± 0.2	9.3 ± 0.8
Δ*Folvps21/FolVPS21*	6.2 ± 0.1	6.1 ± 0.2	3.0 ± 0.2	0.6 ± 0.1	9.6 ± 1.5

*^*a*^Colony diameter of the indicated strains on different media after 5 days incubation at 28°C. ^*b*^Quantification of the Microconidia production of the indicated strains from CMC cultures. ^*c*^Quantification of the Macroconidia production of the indicated strains from CMC cultures. ^*d*^Measurement of the Microconidia length of the indicated strains. ± SD was calculated from three repeated experiments and asterisks indicate statistically significant differences (p < 0.01). NA, not assay.*

Conidia play a key role in infection and subsequent development of pathogenic fungi in the host root (Mes et al., 1999; Li et al., 2019b). Therefore, we investigated the role of FolVps9 in conidia formation. Wild-type, Δ*Folvps9* and Δ*Folvps9/FolVPS9* strains were observed following the induction of conidiophores and conidia formation on carboxymethyl cellulose (CMC) media for 5 days. Microscopic observations showed that conidiophore production by the Δ*Folvps9* mutant was reduced over 50.0% compared with wild type and complemented strains (Figure 1B). Furthermore, compared to wild type, Microconidia was decreased over 60.0% in the Δ*Folvps9* mutant and the conidia produced were smaller (Figure 1C and Table 1). In addition, the macroconidia were reduced 50% in the in the Δ*Folvps9* mutant (Table 1). These results suggest that FolVps9 is important for vegetative growth and conidiogenesis in *Fol*.

### FolVps9 Is Required for *Fol* Virulence in Tomato Roots

**FIGURE 2:**
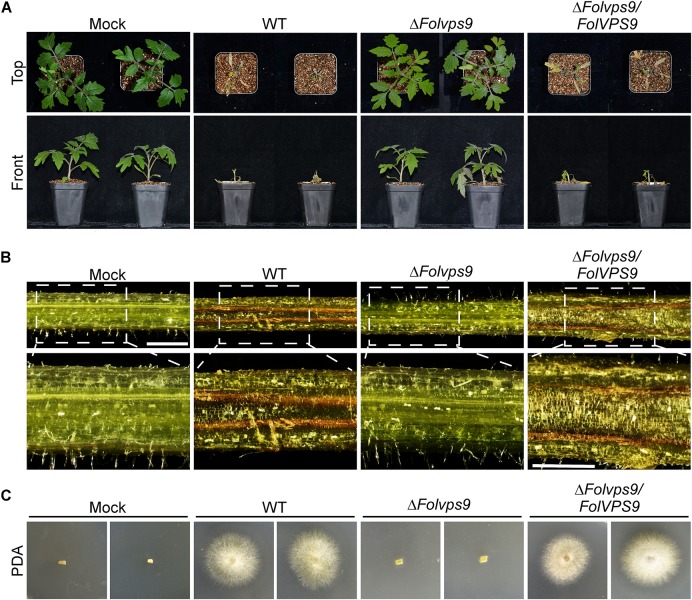
FolVps9 is required for pathogenicity in *Fol*. **(A)** Pathogenicity assays. The susceptible tomato cultivar Moneymaker was inoculated with wild-type, Δ*Folvps9* mutant and complemented strains using a standard root-dip method. Photographs were taken 21 days after infection. **(B)** Vascular discoloration of tomato plants. Tomato plants were inoculated with conidial suspensions and then cultivated for 14 days, after which the stems were split and photographed using a stereo microscope. Bar = 1 mm. **(C)** Outgrowth of fungi from tomato stems of plants inoculated with the indicated strains on PDA medium. Plates were incubated for 2 days.

To assess whether FolVps9 is required for pathogenesis of *Fol*, infection assays were performed using roots from the susceptible cultivar Moneymaker and conidia from wild type, Δ*Folvps9* and Δ*Folvps9/FolVPS9* strains. At 21 dpi, plants inoculated with Δ*Folvps9* showed no obvious disease symptoms and were as healthy as the water-treated (mock) plants (Figure 2A). In contrast, plants treated with the wild type and Δ*Folvps9/FolVPS9* strains exhibited obvious disease symptoms, with severe stunted growth and wilting (Figure 2A).

To gain further insight into the defect of the Δ*Folvps9* mutant on disease progression in tomato, the vascular bundles of plant stems inoculated with conidia of wild type, Δ*Folvps9* and Δ*Folvps9/FolVPS9* strains were assessed using stereomicroscope at 14 dpi. The results showed that the stem vascular bundles of plants inoculated with wild type and Δ*Folvps9/FolVPS9* strains exhibited brown pigmentation, suggesting that the vascular bundles contain lesions, while the color of vascular bundles in the plants infected with the Δ*Folvps9* mutant were similar to those of water-treated control plants (Figure 2B).

Fungal recovery assays were used to further assess the pathogenesis defect of the Δ*Folvps9* mutant. Compared to the wild type and Δ*Folvps9/FolVPS9* strains, infected stems from the Δ*Folvps9* mutant yielded very little fungal growth (Figure 2C). These observations were supported by qPCR fungal biomass quantification results, with significantly higher levels of fungal DNA in the wild type and complemented strains than in the Δ*Folvps9* mutant (Supplementary Figure S2). Taken together, these results indicated that FolVps9 was required for plant infection in *Fol*.

### FolVps9 Is Localized in Endosomes and Functions During Endocytosis

**FIGURE 3:**
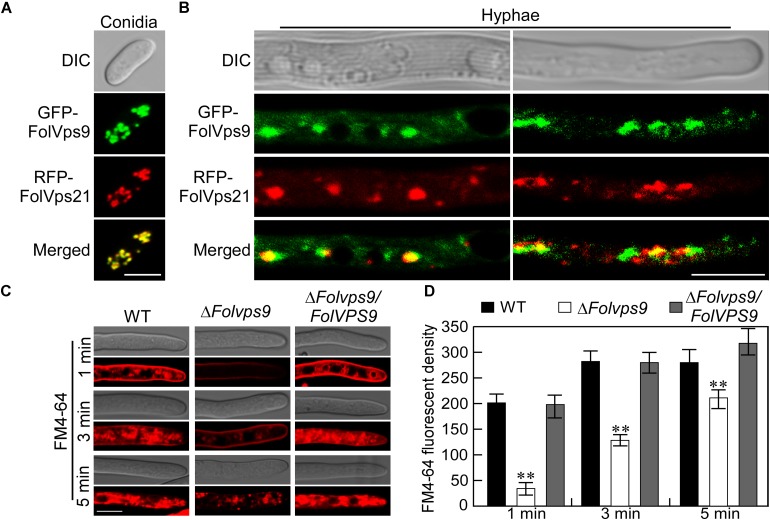
The FolVps9 protein co-localizes with FolVps21 and plays a role in endocytosis. **(A,B)** Conidia and vegetative hyphae expressing the GFP-FolVps9 and RFP-FolVps21 fusion constructs were cultured in liquid CM medium for 24 h. Photographs were taken using differential interference contrast (DIC) and epifluorescence microscopy. Bar = 10 μm. **(C)** Hyphae of indicated strains were grown in liquid CM medium for 24 h and stained with FM4-64 for different times. The camera exposure time was 800 ms. Bar = 10 μm. **(D)** Calculation of FM4-64 relative fluorescence density. Data from experiments described in **(C)** were used for calculations. Error bars represent ± SD and asterisks indicate statistically significant differences (*p* < 0.01).

To further understand the role of FolVps9, we checked its subcellular localization. We constructed an expression cassette for GFP-*FolVPS9* driven by a strong constitutive ribosomal protein promoter, RP27, and transformed into *Fol*. As mentioned above, this construct rescued the phenotypic defects of the Δ*Folvps9* mutant. Fluorescent microscopy of vegetative hyphae from this strain showed that GFP-FolVps9 was localized in punctate cytoplasmic bodies (Figure 3A). Because in yeast Vps9p acts as a GEF for Vps21p, a protein involved in vesicle trafficking and a marker protein for early endosomes, we speculated that the punctuate bodies in *Fol* may contain the homolog of Vps21, FolVps21. To test this hypothesis, we constructed a RFP-*FolVPS21* expression vector and co-transformed with the GFP-FolVps9 construct into *Fol*. The resulting neomycin-resistant transformants were screened by detection of the GFP and RFP signals using fluorescence microscopy. We found that a large proportion of the GFP-FolVps9 and RFP-FolVps21 proteins were co-localized in conidia and hyphae (Yellow signal; Figures 3A,B). This result suggested that FolVps9 was at least partially localized in endosomes.

The co-localization of FolVps9 with an endosomal marker such as FolVps21 indicates that it may function during endocytosis. To determine whether FolVps9 is involved in endocytosis, we using FM4-64, a fluorescent dye that stains phospholipids and has been used to track endocytosis in fungi, to investigate endocytosis in the Δ*Folvps9* mutant (Fischer-Parton et al., 2000; Qi et al., 2016). In the wild-type and complemented strains, the FM4-64 stain was gradually internalized and stained endosomes were visible 1 min after incubation (Figures 3C,D). In contrast, only a weak fluorescent signal was visible on the plasma membrane in the Δ*Folvps9* mutant after 1 min incubation, and endosomal compartments were not stained until 5 min (Figures 3C,D). These results suggested that endocytosis was delayed in the Δ*Folvps9* mutant.

### The Vps9 Domain of FolVps9 Is Required for Its Normal Localization and Function

**FIGURE 4:**
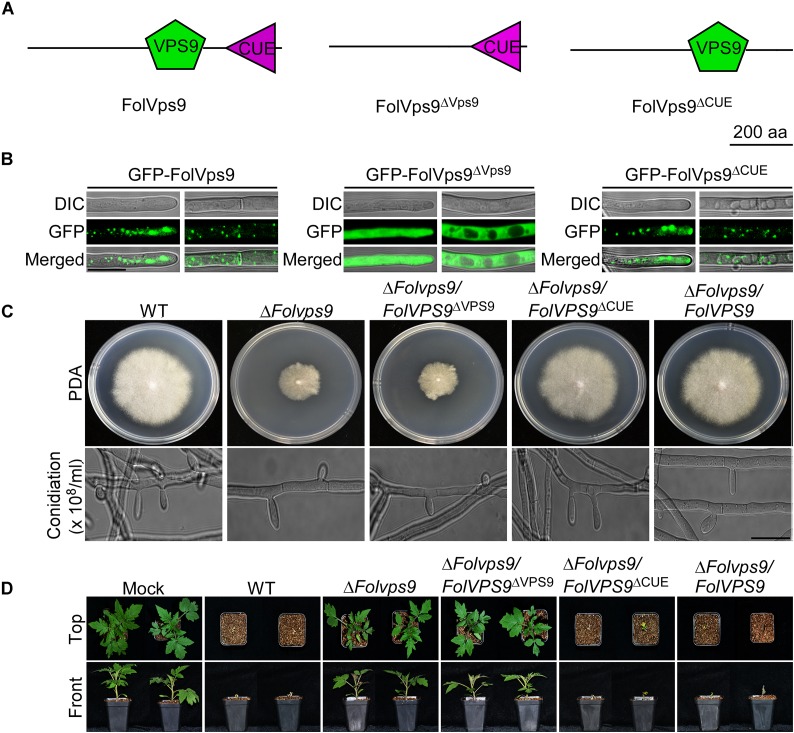
Functional characterization of the Vps9 and Cue domains in FolVps9. **(A)** Schematic showing the position of the Vps9 and/or Cue domains in the three FolVps9 alleles. **(B)** Subcellular localization of the mutated FolVps9 proteins. Hyphae were imaged as described in Figure 3. **(C)** Growth and conidiophore formation analysis were performed as described in Figure 1. Bar = 10 μm. **(D)** Pathogenicity assays were performed as described in Figure 2 and photographs were taken at 21 dpi.

To investigate the contribution of each domain (Vps9 and Cue) of FolVps9 to subcellular localization and function, we generated GFP-FolVps9^ΔVps9^ and GFP-FolVps9^ΔCue^ constructs lacking the Vps9 and Cue domain, respectively, and transformed each construct into the Δ*Folvps9* mutant (Figure 4A). The GFP signal of FolVps9^ΔCue^ was localized to punctate structures (presumptive endosomes), similar to GFP-FolVps9 (Figure 4B). However, the green fluorescence of FolVps9^ΔVps9^ was distributed throughout the cytoplasm (left) and excluded from vacuoles (right) (Figure 4B).

Next, we assayed possible functions of the Vps9 and Cue domains in fungal development and plant infection. In contrast to the wild type and Δ*Folvps9/FolVPS9* strains, FolVps9^ΔVps9^ exhibited phenotypes similar to the Δ*Folvps9* mutant, whereas FolVps9^ΔCue^ did not possess obvious defects (Figures 4C,D and Table 1). These results suggest that the Vps9, but not the Cue, domain is required for the correct localization and physiological functions of FolVps9.

### FolVps9 Is Essential for Autophagy in *Fol*

During the process of autophagy, the autophagosomes are transported to vacuoles for degradation via vesicle trafficking. The autophagy process can be analyzed by monitoring the vacuolar delivery and subsequent breakdown of GFP-Atg8 by vacuolar hydrolases (Liu et al., 2015). To test whether *FolVPS9* plays a role in autophagy, we introduced the autophagy marker fusion gene GFP-*FolATG8* into wild type and the Δ*Folvps9* mutant. We analyzed the strains for autophagy flux using epifluorescence microscopy and also examined the autophagy bodies of hyphal cells grown in rich medium (CM) or minimal medium without NaNO_3_ (MM-N) by transmission electron microscopy.

**FIGURE 5:**
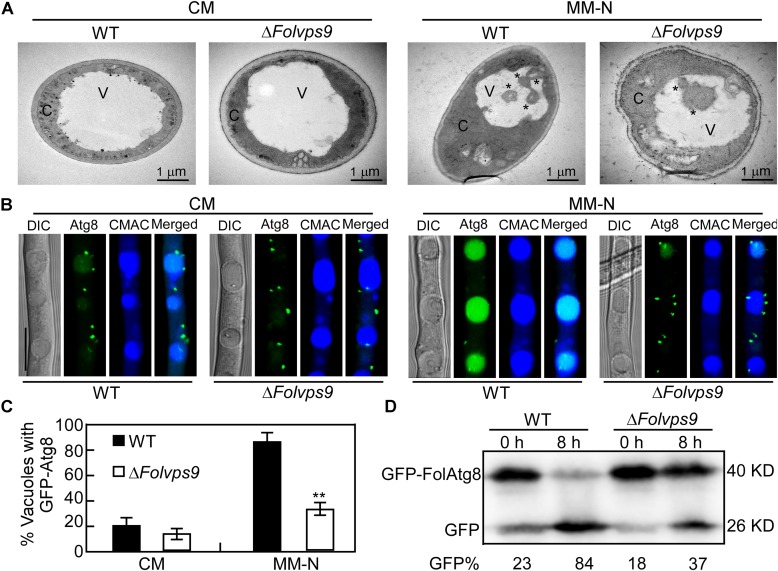
Autophagy defects in the Δ*Folvps9* mutant. **(A)** Observation of organelles and autophagic bodies using transmission electron microscopy. Mycelia of the indicated strains were cultures in liquid CM medium at 28°C for 16 h and then shifted to liquid MM-N medium containing 4 mM phenylmethylsufonyl fluoride (PMSF) for 8 h. Bar = 1 μm. C, cytoplasmic; V, vacuole. Asterisks indicate autophagosomes. **(B)** Localization of GFP-FolAtg8 in wild type and Δ*Folvps9* mutant backgrounds. Wild type and Δ*Folvps9* mutant strains expressing GFP-FolAtg8 were grown in liquid CM medium at 28°C for 16 h and shifted to liquid MM-N medium containing 4 mM PMSF for 8 h. Mycelia were stained with CMAC and examined under using DIC or fluorescence microscopy. Bar = 10 μm. **(C)** Statistical analysis for autophagy of 100 cells from wild type and the Δ*Folvps9* mutant **(B)** was performed as described above. Error bars represent ± SD and asterisks indicate statistically significant differences (*p* < 0.01). **(D)** GFP-FolAtg8 proteolysis assays in wild type and the Δ*Folvps9* mutant. Protein was extracted from mycelia cultured in liquid CM medium for 16 h ± induction in MM-N for 8 h, and analyzed by western blot using anti-GFP antibody. The extent of autophagy was estimated by calculating the amount of free GFP compared with the total amount of intact GFP-FolAtg8 and free GFP (the amounts are shown beneath the blot).

When grown in CM rich medium for 16 h, no autophagy bodies or GFP-FolAtg8 fluorescence signals appear in punctuate structures, and the surrounding CMAC stained vacuoles are distributed throughout the cytoplasm of the wild-type and Δ*Folvps9* mutant strains (Figures 5A–C). When hyphae were shifted to nitrogen-starvation conditions (MM-N) for another 8 h, numerous autophagy bodies and GFP fluorescence signals accumulated in the vacuoles of wild type (Figures 5A–C). However, little GFP fluorescence signal and few autophagy bodies were observed in the vacuoles of the Δ*Folvps9* mutant strain (Figures 5A–C).

In order to further verify these observations and for a more systematic evaluation, we also performed a GFP-FolAtg8 proteolysis assay. Under normal conditions (CM), a band corresponding to full-length GFP-FolAtg8 fusion protein (40 kDa) and a weaker free GFP band (26 kDa) could be readily detected in both wild type and the Δ*Folvps9* mutant using an anti-GFP antibody (Figure 5D). When hyphae were shifted to nitrogen-starvation conditions (MM-N) for 8 h, wild type exhibited reduced levels of full-length GFP-FolAtg8 and a more abundant free GFP band (Figure 5D). In contrast, compared to wild type, levels of full length GFP-FolAtg8 were relatively greater in the Δ*Folvps9* mutant on MM-N medium (Figure 5D). Thus, the GFP-FolAtg8 assays demonstrated that FolVps9 is required for normal GFP-FolAtg8 proteolysis. Taken together, our results suggest that FolVps9 is important for autophagy in *Fol*.

### FolVps9 Interacts With the GDP-Bound Form of FolVps21

In order to screen for potential regulators of endocytosis and autophagy in response to FolVps9, we used the FolVps9-3 X *FLAG* strain to identify co-immunoprecipitated (IP) proteins by mass spectrometry (Table 2). Proteins that co-immunoprecipitated with FolVps9 include the Rab GTPase FolYpt1 and a number of vesicle trafficking proteins (Table 2). One of the FolVps9 interacting proteins, FolVps21, is highly similar to yeast Vps21p that functions in endocytosis and autophagy. Vps21 is a small Ras-related GTPase that can continuously cycle between the cytosol and plasma membrane by converting between its GDP-bound and GTP-bound form (Krick et al., 2008). Several *vps21* mutations that lead to constitutive occupancy with GDP or GTP have been well studied in yeast and *M. oryzae*: for example, the S21L/S25N mutation (GDP-bound form) and Q66L/Q70L mutation (GTP-bound form) in yeast and *M. oryzae*, respectively (Lo et al., 2011; Zhu et al., 2018). Based on this precedent, we examined wild type FolVps21 and the FolVps21^S27N^ (predicted GDP-bound form) and FolVps21^Q72L^ (predicted GTP-bound form) mutations.

**TABLE 2:** Putative FolVps9-interacting proteins related to endocytosis and autophagy identified by affinity purification.

**Gene ID**	**Description**	**Homology** **in yeast**	**Unique** **peptides**	**MW** **(kDa)**
FOXG_02298	Small COPII coat GTPase	Sar1	2	21.5
FOXG_03079	Myosin heavy chain	Myo1	4	275.5
FOXG_12799	Syntaxin 7	Vam3	2	29.7
FOXG_09392	Rab family GTPase	Vps21	3	24.7
FOXG_03741	Golgi transport complex subunit	Cog5	2	48.5
FOXG_12808	GTP-binding protein ypt1	Ypt1	3	22.4
FOXG_07785	Hypothetical protein	Vps41	4	148.9
FOXG_04244	Syntaxin 16	Tlg2	3	38.6
FOXG_02138	Hypothetical protein	Muk1	2	76.8

**FIGURE 6:**
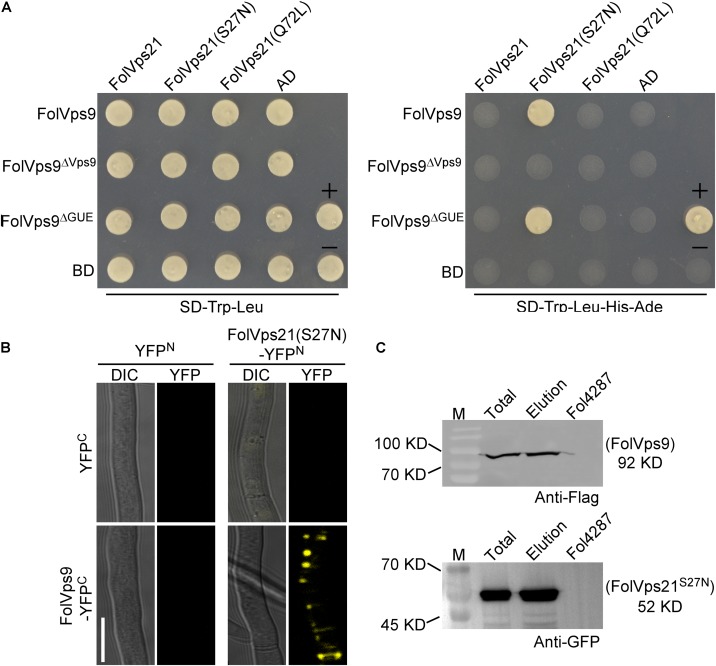
FolVps9 interacts with the predicted GDP-bound form of FolVps21. **(A)** Yeast two-hybrid assays. Yeast cells expressing the prey and bait constructs were assayed for growth on SD-Leu-Trp and SD-Leu -Trp-His-Ade plates. **+**, positive control; -, negative control. **(B)** Bimolecular fluorescence complementation (BiFC) analysis. Mycelia of transformants expressing the FolVps9-YFP^C^/FolVps21^S27N^-YFP^N^ constructs were observed by DIC or fluorescence microscope. Bar = 10 μm. **(C)** Co-immunoprecipitation assays. Total proteins were extracted from transformants co-expressing FolVps9-3xFLAG and GFP-FolVps21^S27N^ constructs, and protein eluted from the anti-GFP M2 beads (elution). Immunoblots were incubated with monoclonal anti-FLAG or anti-GFP antibody, as indicated.

We first used the yeast two-hybrid (Y2H) assay to detect interactions between FolVps9 and the three states of FolVps21. The Y2H results showed that FolVps9 specifically interacted with FolVps21^S27N^, but not with FolVps21 or FolVps21^Q72L^ (Figure 6A), indicating that FolVps9 might act as a FolVps21 GEF. We next explored the role of each domain (Vps9 and Cue) of FolVps9 in the interaction with FolVps21, by generating Y2H constructs expressing FolVps9^Δ^ ^Vps9^ and FolVps9^Δ^ ^Cue^ and assaying their interaction with FolVps21^S27N^ by Y2H. The results revealed that the Vps9 domain was required for the interaction of FolVps9 with FolVps21^S27N^ (Figure 6A).

The Y2H interaction between FolVps9 and FolVps21^S27N^ was corroborated using BiFC and co-IP assays in *Fol* (Figures 6B,C). Fluorescence was only observed in a *Fol* strain expressing both FolVps9-YFP^C^ and FolVps21^S27N^-YFP^N^ (Figure 6B). Taken together, these results indicate that FolVps9 interacts with FolVps21^S27N^, but not with wild type FolVps21 or the GTP-bound form, FolVps21^Q72L^.

### FolVps21 Is Important for Growth Polarity, Conidiation, and Full Virulence

**FIGURE 7:**
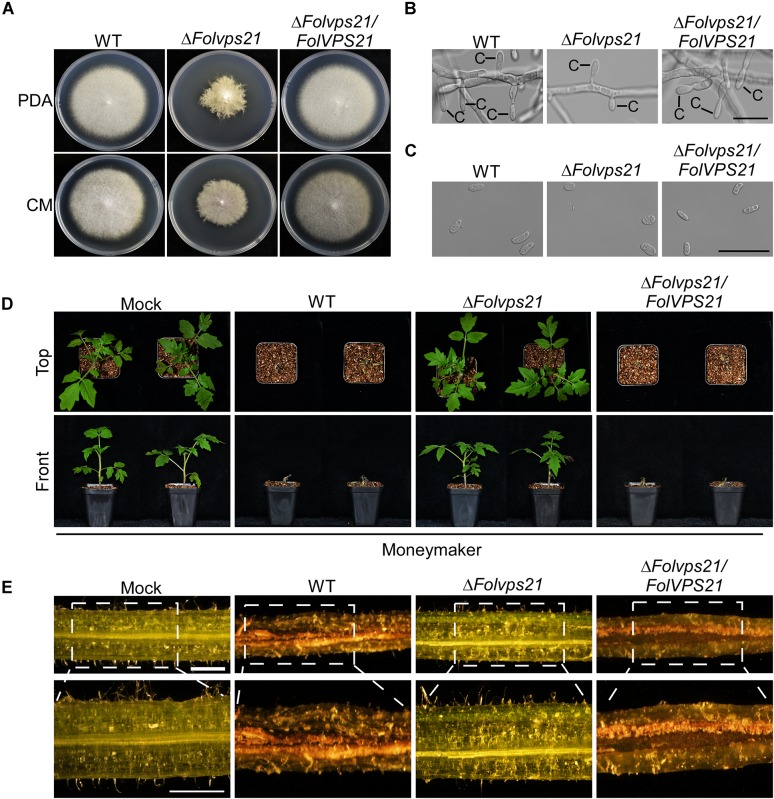
FolVps21 is important for growth polarity, asexual development and full virulence. **(A)** Colony growth and morphology assays were performed as described in Figure 1. **(B)** Conidiophore development assays were performed as described in Figure 1. Bar = 10 μm. C, conidium. **(C)** Conidial morphology. Conidia from wild-type, Δ*Folvps21* mutant and the complemented strains were observed by DIC microscopy. Bar = 5 μm. **(D)** Moneymaker tomato plants were infected with the indicated strains as described in Figure 2 and photographs were taken at 21 dpi. **(E)** Vascular discoloration. Tomato plants were inoculated with conidial suspensions for 14 days, after which the stems were split and photographed using a stereomicroscope. Bar = 1 mm.

Because FolVps21 physically interacts with FolVps9, we further characterized the biological role of *FolVPS21* using a targeted gene replacement strategy in *Fol* (Supplementary Figures S1A,C). The Δ*Folvps21* gene deletion mutant displayed very similar phenotypes to the Δ*Folvps9* mutant. The polarized growth rate was slower in the Δ*Folvps21* mutant (Figure 7A and Table 1). Microscopic observations showed that the ability to produce conidia was reduced in the Δ*Folvps21* mutant (Figure 7B). After culturing in CMC medium for 5 days, the Δ*Folvps21* mutant produced 1.5 × 10^8^ Micconidia/ml compared to 3.1 × 10^8^ microconidia/ml produced by the wild-type strain (Table 1). Furthermore, 78.3% of the conidia produced by the Δ*Folvps21* mutant were smaller than those from the wild type (Table 1). In addition, the Δ*Folvps21* mutant exhibited reduced macroconidia and virulence on tomato (Table 1 and Figures 7D,E, Supplementary Figure S2). The defects in development and pathogenicity were fully restored in the complemented strain (Figure 7 and Table 1). Taken together, these results suggest that FolVps9 and FolVps21 function together in mediating the same physiological process in *Fol*.

### Constitutively Activated FolVps21 Rescues the Defects of the Δ*Folvps9* and Δ*Folvps21* Mutants

**FIGURE 8:**
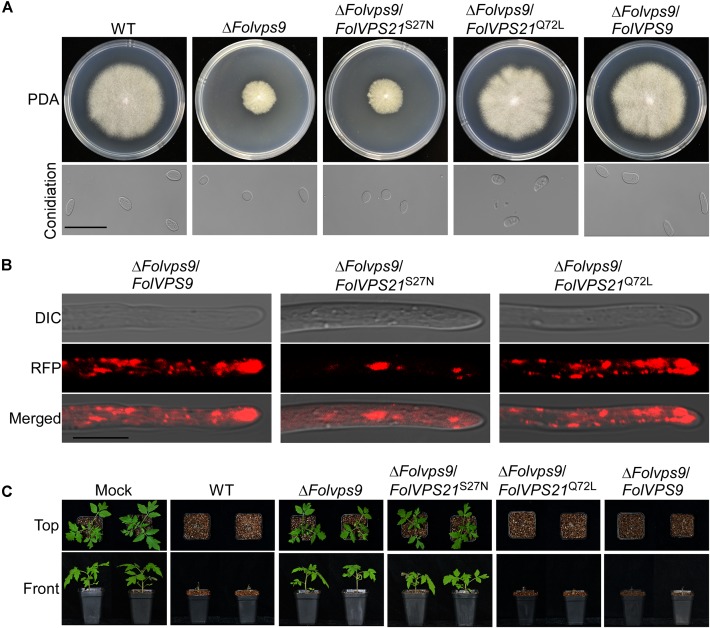
Constitutively activated FolVps21 (Q72L) rescues the defects of the Δ*Folvps9* mutant strain. **(A)** Colony and conidiation morphology assays were performed as described in Figure 1. Bar = 10 μm. **(B)** Subcellular localization of the indicated different states of the FolVps21 protein. Bar = 10 μm. **(C)** Pathogenicity assays. Tomato seedlings were inoculated with conidial suspensions from the indicated strains as described in Figure 2 and photographs were taken at 21 dpi.

Numerous studies in yeast suggest that Vps9p functions as highly efficient GEF and positive regulator for Vps21p (Esters et al., 2001; Bean et al., 2015; Zhu et al., 2018). Our results demonstrating that FolVps9 only interacts with the predicted GDP-bound form of FolVps21 and that mutants lacking either gene possess the same phenotypes supports a similar scenario in *Fol*. To further investigate whether FolVps9 might function as a positive regulator/GEF for FolVps21 in *Fol*, we generated constitutively active *FolVPS21*^Q72L^ (GTP-bound form) and dominant negative *FolVPS21*^S27N^ (GDP-bound form) constructs under the control of the RP27 promoter and transformed them into protoplasts of the Δ*Folvps9* mutant. The resulting Δ*Folvps9*/*FolVPS21*^Q72L^ transformants were examined expression of FolVps21 by qRT-PCR analysis (Supplementary Figure S3). Phenotypic analysis revealed that the Δ*Folvps9*/*FolVPS21*^Q72L^ transformant displayed normal vegetative growth, conidiation, conidial morphology, subcellular localization and virulence compared to wild type (Figure 8 and Table 1). In contrast, the Δ*Folvps9*/*FolVPS21*^S27N^ strain resembled the Δ*Folvps9* mutant. These results support a positive role for FolVps9 in regulating the biological function of FolVps21, consistent with a function as a GEF for FolVps21.

**FIGURE 9:**
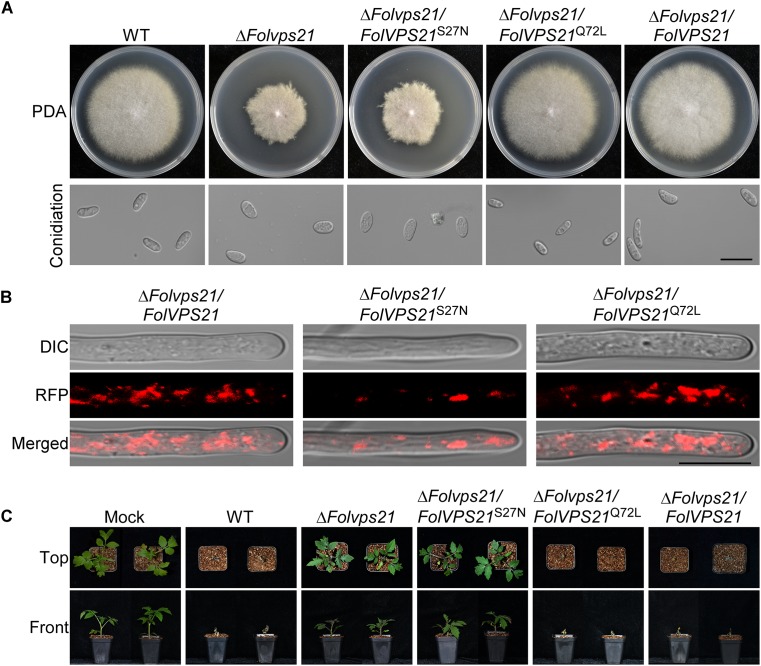
Constitutively activated FolVps21 (Q72L) rescues the defects of the Δ*Folvps21* mutant strain. **(A)** Colony and conidial morphology assays were performed as described in Figure 1. Bar = 10 μm. **(B)** Subcellular localization of the indicated different states of the FolVps21 protein. Bar = 10 μm. **(C)** Pathogenicity of mutants in tomato. Moneymaker tomato seedlings were inoculated with conidial suspensions of the indicated strains as described in Figure 2 and photographs were taken at 21 dpi.

We also transformed *FolVPS21*^S27N^ and *FolVPS21*^Q72L^ constructs into protoplasts of the Δ*Folvps21* mutant. The resulting Δ*Folvps21*/*FolVPS21*^Q72L^ transformants were examined by qRT-PCR analysis (Supplementary Figure S3). Phenotypic analysis gave results very similar to those observed for the *FolVPS21*^S27N^ and *FolVPS21*^Q72L^ constructs transformed into the Δ*Folvps9* mutant. The data revealed that growth, conidiation and pathogenicity defects associated with the Δ*Folvps21* mutation were fully rescued by *FolVPS21*^Q72L^ (Figure 9 and Table 1). However, the *FolVPS21*^S27N^ allele was not able to rescue the phenotype defects of the Δ*Folvps21* mutant (Figure 9 and Table 1). In addition, the localization pattern of RFP-tagged FolVps21^Q72L^ in the Δ*Folvps21*/RFP-*FolVPS21*^Q72L^ strain was similar to that observed in the Δ*Folvps21*/RFP-*FolVPS21* strain (Figure 9). The localization pattern of RFP-tagged FolVps21^Q72L^ in the Δ*Folvps21*/RFP-*FolVPS21* ^S27N^ strain was altered compared to that of the wild type protein observed in the Δ*Folvps21*/RFP-*FolVPS21* strain (Figure 9). Taken together, these results showed that constitutively activated FolVps21 can rescue the growth, developmental and pathogenicity defects of the Δ*Folvps9* and Δ*Folvps21* mutants in *Fol*.

### Constitutively Activated *FolVps21* Corrects the Endocytosis and Autophagy Defects of the Δ*Folvps9* Mutant

**FIGURE 10:**
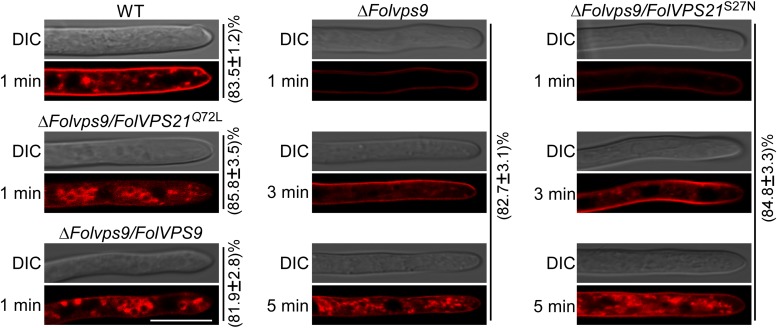
Constitutively activated FolVps21 (Q72L) corrects the endocytosis defects of the Δ*Folvps9* mutant strain. Strains were grown in liquid CM medium for 48 h, then stained with FM4-64 and examined using a confocal fluorescence microscope (Zeiss LSM710, 63x oil objective) as described in Figure 3. The camera exposure is indicated in milliseconds (800 ms). The numbers indicate the percentage of hyphae exhibiting the endocytosis pattern seen in the figure (*n* = 100). Bar = 10 μm.

As shown above, phenotypic data indicates that constitutively FolVps21 can rescue Δ*Folvps9* defects. In order to test the effects of constitutively activated FolVps21 on endocytosis impairments in the Δ*Folvps9* mutant, we performed FM4-64 dye uptake experiments. The defects in FM4-64 uptake by the Δ*Folvps9* mutant were suppressed by expression of constitutively activated FolVps21, but not by constitutively inactive FolVps21 (Figure 10). The Δ*Folvps9*/*FolVPS21*^Q72L^ (85.8%) strain exhibited relatively faster FM4-64 uptake (1 min) than the Δ*Folvps9* mutant (5 min), while FM4-64 uptake in the Δ*Folvps9*/*FolVPS21*^S27N^ (84.8%) strain was similar to the Δ*Folvps9* mutant (5 min) (Figure 10). These results suggest that constitutively activated FolVps21 suppresses the endocytosis defects of the Δ*Folvps9* mutant.

**FIGURE 11:**
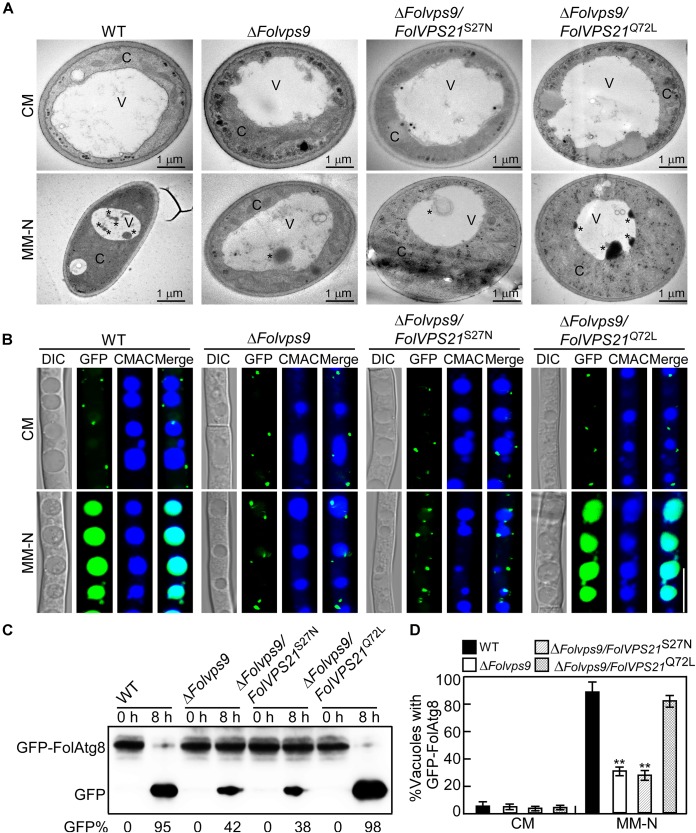
Constitutively activated FolVps21 (Q72L) rescues the autophagy defects of the Δ*Folvps9* mutant strain. **(A)** Examination of organelles and autophagic bodies by transmission electron microscopy as described in Figure 5. Bar = 1 μm. C, cytoplasmic; V, vacuole. Asterisks indicate autophagosomes. **(B)** Localization of GFP-FolAtg8 and staining with 7-amino-4-chloromethylcoumarin (CMAC). The indicated Fol strains were assayed as described in Figure 5. Bar = 10 μm. **(C)** GFP-FolAtg8 proteolysis assays were performed as described in Figure 5. **(D)** Statistical analysis of 100 cells from the indicated strains **(B)** for GFP signal in the vacuole. Error bars represent ± SD and asterisks indicate statistically significant differences (*p* < 0.01).

We also utilized transmission electron microscopy to observe autophagy bodies of hyphal cells in our strains. When grown in CM medium, no autophagy bodies were observed in the hyphae of any strain (Figure 11A). However, when hyphae were shifted to MM-N medium, numerous autophagy bodies accumulated in the vacuoles of wild-type and Δ*Folvps9/FolVPS21*^Q72L^ strains (Figure 11A).

We next analyzed the GFP-FolAtg8 fluorescence signals under two culture conditions in our strains. In CM medium, the GFP-FolAtg8 fluorescence signal existed in punctuate structures and the surrounding CMAC-stained vacuoles were distributed in the cytoplasm of all strains (Figure 11B). After culture in MM-N medium, GFP fluorescence signals accumulated in the vacuoles of wild-type and Δ*Folvps9/FolVPS21*^Q72L^ strains (Figure 11B). However, little GFP fluorescence and few autophagy bodies were observed in the vacuoles of the Δ*Folvps9* mutant and Δ*Folvps9/FolVPS21*^S27N^ strains (Figures 11B,D). To further confirm these observations, we also performed a GFP-FolAtg8 proteolysis assay. Under normal conditions, a clear full length GFP-FolAtg8 fusion protein band (40 kDa) could be detected in all strains (Figure 11C). When hyphae were shifted to nitrogen-starvation conditions, the wild-type and Δ*Folvps9/FolVPS21*^Q72L^ strains displayed relatively weak full-length GFP-FolAtg8 and an abundant GFP band (Figure 11C). Compared to wild type, levels of full length GFP-FolAtg8 did not significantly decrease in the Δ*Folvps9* and Δ*Folvps9/FolVPS21*^S27N^ strains (Figure 11C). These results support the hypothesis that constitutively activated FolVps21 can rescue autophagy defects of the Δ*Folvps9* mutant.

## Discussion

**FIGURE 12:**
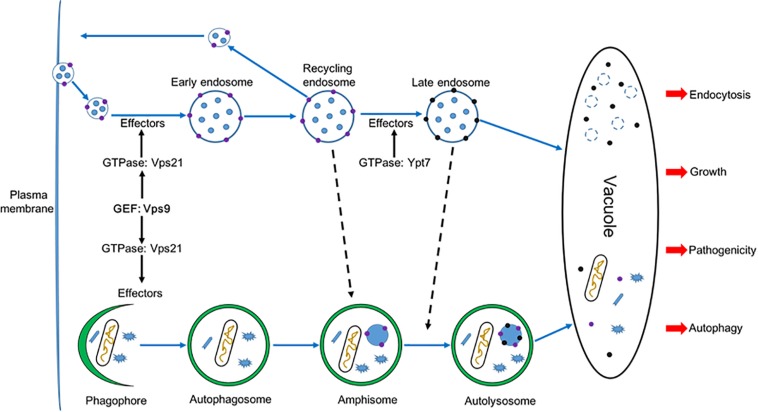
A proposed model for FolVps9 during the development, plant infection, autophagy and endocytosis in *Fol*. Phagophore: A double membrane that encloses and isolates the cytoplasmic components during macroautophagy; Autophagosome: a spherical structure with double layer membranes; Amphisome: An autophagic vacuole formed by fusion of an autophagosome and an endosome; Autolysosome: a vacuolar element of the lysosome system of cells to which hydrolases have been added by fusion with lysosomes.

Guanine nucleotide exchange factors (GEFs) facilitate release of GDP and the binding of GTP for subsequent activation of Rab GTPases (Ishida et al., 2016). The Vps9 GEF protein and the Vps21 GTPase have been reported to play important roles in modulating development in organisms from yeast to humans (Esters et al., 2001; Paulsel et al., 2013). However, the autophagy and endocytosis functions of Vps9 protein have been investigated in a limited number of studies in filamentous fungi, and with only one plant fungal pathogen (*M. oryzae*). In this study, we have identified and characterized the Vps9 homolog FolVps9 in the tomato pathogen *Fol* and showed that FolVps9 was required for fungal growth, development, endocytosis, autophagy and plant pathogenicity. Based on genetic evidence and cytological examination, we further obtained evidence supporting a role for FolVps9 as a GEF for FolVps21, by revealing a physical association between the two proteins and showing that constitutively activated FolVps21 was able to rescue the defects of the Δ*Folvps9* mutant. In addition, we demonstrated that FolVps21 is also important for fungal growth, development and plant infection, and that the FolVps9-FolVps21^Q72L^ interaction plays an important role in endocytosis and autophagy of *Fol* (Figure 12).

In this study, we determined that the Δ*Folvps9* mutant had striking defects in polarity growth, conidiogenesis and pathogenicity. Consistent with these results, in *F*. *graminearum*, Vps39, a HOPS complex subunit, localizes to early endosomes (EEs) and plays key roles in endocytosis and morphology (Li B. et al., 2017). Other studies have shown that endocytosis was important for the pathogenic development of different plant fungal pathogens (Li L. et al., 2017; Li X. et al., 2017; Li et al., 2019b). Through systems-level analysis of the functions of endocytic proteins, such as the early endocytic protein MoEnd3 (Li X. et al., 2017), the SNARE protein FolVam7 (Li et al., 2019b), and the capping protein MoCap (Li L. et al., 2017), it was revealed that endocytosis was important for development and virulence in different plant fungal pathogens.

Autophagy is a fundamental function in eukaryotes and is well conserved from mammals to yeasts. Nevertheless, there is a little evidence to demonstrate any relationship between endocytosis and autophagy in filamentous fungi. Here, we found that FolVps9 co-localized with FolVps21 and that the Δ*Folvps9* mutant exhibited defects in endocytosis. In addition, the Δ*Folvps9* mutant has defects in the trafficking and degradation of autophagosomes. Several Rab proteins were well-known mediators of vesicle trafficking and involved in regulation of autophagy (Liu et al., 2015; Zheng et al., 2018b). For example, Rab5 plays roles in vesicle trafficking, cargo recycling and maturation of early endosomes and is involved in membrane fusion events. Similar to its endosome function, Rab5 can recruit the autophagic BECN1-PIK3C3 complex and production PI3P on early stage of autophagosome formation (Ravikumar et al., 2008). Rab1 regulates vesicle trafficking and functions during early autophagy (Zoppino et al., 2010).

In *Fol*, we did not able to isolate the Δ*Folvps9* and Δ*Folvps21* double mutant and it was thus difficult to directly demonstrate the relationship between autophagy and endocytosis. However, based on our functional analysis of FolVps9 and previous work, we speculate that there is crosstalk between autophagy and endocytosis. Here, we used mass spectrometry to identify several candidate vesicle trafficking and Rab GTPase proteins that interact with FolVps9, including FolYpt1, FolVps21, FolSar1, FolVam3, and FolVps41. Furthermore, our protein-protein interaction results confirmed that FolVps9 interacts with the GDP-bound form of FolVps21. Taken together, these results suggest that FolVps9 interacts with these candidate proteins to participate in autophagy and endocytosis in *Fol*.

The reduced virulence of the Δ*Folvps9* mutant results from multiple defects. First, the vegetative growth of Δ*Folvps9* mutant was significantly reduced relative to the wild type strain. Consistent with these results, the decreased growth of the Δ*Folvps9* mutant led to attenuated virulence in the plant pathogenic fungus *M. oryzae* (Zhu et al., 2018). Second, the Δ*Folvps9* mutant showed delayed endocytosis, which was regarded as one of fungal responses to plant infection (Li X. et al., 2017; Li B. et al., 2017). We obtained evidence that the impaired virulence of the Δ*Folvps9* mutant was due to a defect in endocytosis, in that the impairment in virulence was suppressed when endocytosis was rescued in the Δ*Folvps9* mutant. Third, we showed that perturbing the autophagy pathway causes attenuated virulence in the Δ*Folvps9* mutant, as the autophagy pathway is well conserved and important during the invasion of host cells (Kershaw and Talbot, 2009; Lv et al., 2017). Following the constitutive activation of *FolVps21* in the Δ*Folvps9* mutant, defects in autophagy and virulence were suppressed, indicating that autophagy was important to infection for *Fol*.

Insights from a previous study indicate that the Vps9 domain is important for regulating protein-protein interactions in bacteria (Bair et al., 2008). In this study, we demonstrated that the Vps9 domain was required for the interaction between FolVps9 and FolVps21^S27N^. This result was consistent with what has been observed in *M*. *oryzae* and *F*. *graminearum* (Li et al., 2015; Zheng et al., 2018a; Zhu et al., 2018). More importantly, we found that while expression of constitutively active FolVps21 in the *FolVPS9* deletion mutant could restore normal development and pathogenesis, the dominant negative form of FolVps21 could not rescue the defects associated with the Δ*Folvps9* mutant. Taken together, our results indicate that FolVps9 likely functions as a GEF for FolVps21 to convert FolVps21-GDP to the GTP-bound form to promote vegetative growth, development and infection in *Fol*.

In summary, we identified and characterized *FolVPS9*, a gene encoding a putative vacuolar sorting protein in *Fol*, which plays crucial role in hyphal growth, conidiation, endocytosis, autophagy and pathogenesis. We also provide evidence that FolVps9 might function as a GEF for FolVps21. Our studies provide preliminary evidence for crosstalk between autophagy and endocytosis in *Fol*. Further research efforts will be focused on investigation of the precise molecular function of FolVps9 and its associated proteins and how they regulate endocytosis and autophagy. Results from our studies also provide targets for developing sustainable control strategies for fungal plant pathogens.

## Data Availability Statement

All datasets generated for this study are included in the article/Supplementary Material.

## Author Contributions

S-QO and BL conceived and designed the experiments and wrote the manuscript. BL, H-YM, and Z-YZ performed the experiments. BL, X-JC, and S-QO analyzed the experiment data. All authors have read and approved the final manuscript.

## Conflict of Interest

The authors declare that the research was conducted in the absence of any commercial or financial relationships that could be construed as a potential conflict of interest.

## Footnotes

1 http://eupathdb.org/eupathdb/

2 http://smart.embl-heidelberg.de/

## Supplementary Material

FIGURE S1 Generation of the *FolVPS9* and *FolVPS21* deletion mutants. **(A)** Schematic diagram of the *FolVPS9* and *FolVPS21* genes, and split-marker gene replacement constructs. HPH-N and HPH-C are the N and C-terminal halves of the cassette that confers resistance to hygromycin B. **(B,C)** Southern blot analysis. Genomic DNA from wild type and presumptive deletion mutants was digested with *Eco*RI and *Hin*dIII subjected to Southern blot analysis using gene-specific and *HPH* probes, respectively. WT, Wild type.

FIGURE S2 qPCR to determine relative levels of *Fol* in stem sections of inoculated tomato plants. Genomic DNA was isolated from tomato stems of the Moneymaker cultivar infected with various *Fol* strains as described in section Materials and Methods. Quantitative PCRs were performed to evaluate the fungal biomass using primers that amplify the intergenic spacer region of the ribosomal 28S subunit. The mean values of three determinations with standard deviations are shown. Asterisks indicate statistically significant difference relative to wild type *Fol* (*p* < 0.01).

FIGURE S3 Expression of *FolVPS21* in the indicated strains. The expression of *FolVPS21* was measured by quantitative real-time PCR with cDNA isolated from hyphae of the indicated strains. Levels were first normalized to actin. The relative abundance of *FolVPS21* transcripts was then normalized to that in wild-type vegetative hyphae (Relative transcript level = 1). Error bars represent ± SD and asterisks indicate statistically significant differences (*p* < 0.01).

TABLE S1 Wild-type and mutant strains of fungi used in this study.

TABLE S2 Primers used in this study.
