# Supplementary figures and images for: FolVps9, a Guanine Nucleotide Exchange Factor for FolVps21, Is Essential for Fungal Development and Pathogenicity in Fusarium oxysporum f. sp. lycopersici
Source: Front Microbiol. 2019 Nov 14;10:2658. doi: 10.3389/fmicb.2019.02658 (PMC6868059; doi:10.3389/fmicb.2019.02658)

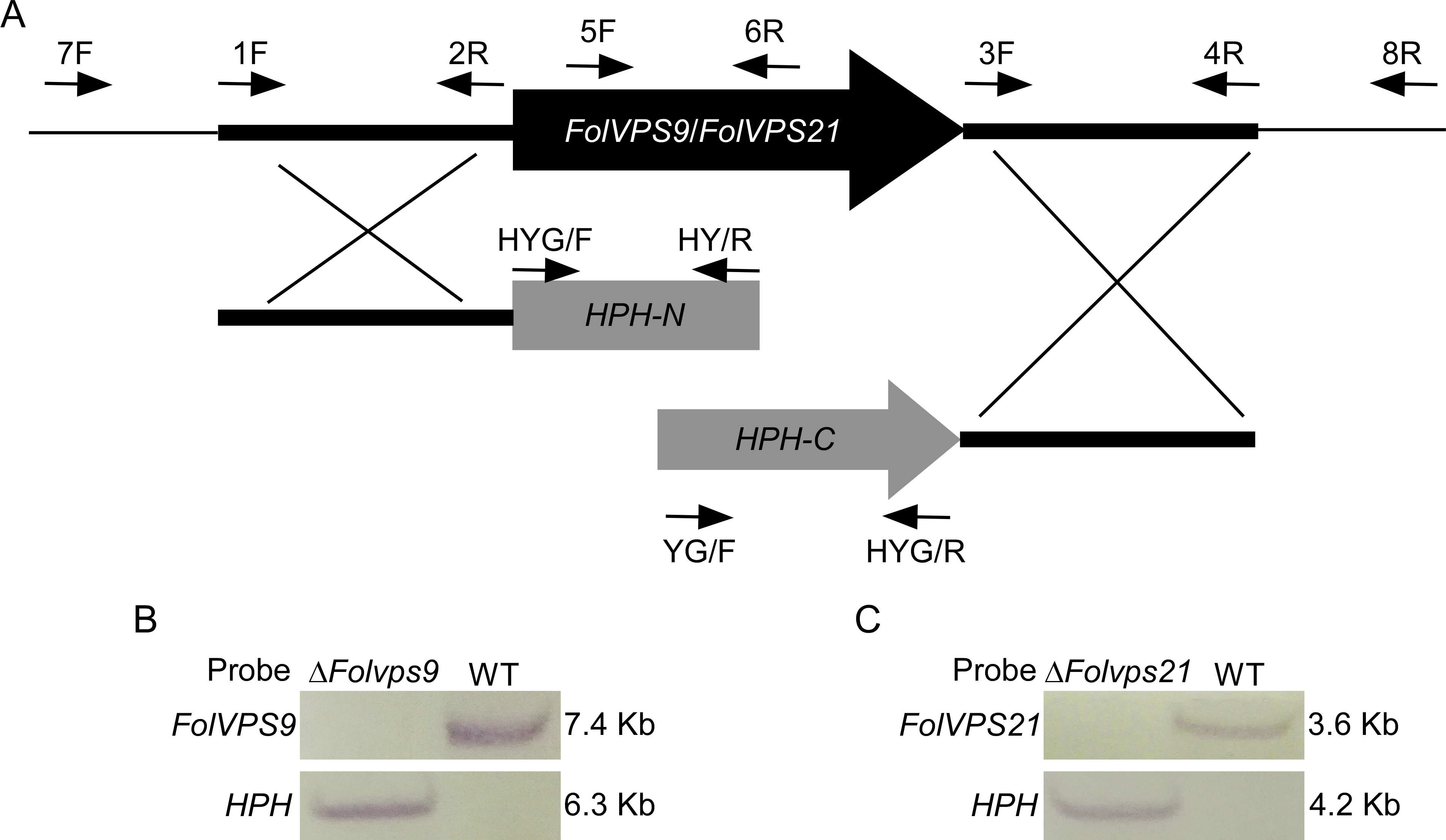

Supplement: FIGURE S1 — Generation of the FolVPS9 and FolVPS21 deletion mutants. (A) Schematic diagram of the FolVPS9 and FolVPS21 genes, and split-marker gene replacement constructs. HPH-N and HPH-C are the N and C-terminal halves of the cassette that confers resistance to hygromycin B. (B,C) Southern blot analysis. Genomic DNA from wild type and presumptive deletion mutants was digested with EcoRI and HindIII subjected to Southern blot analysis using gene-specific and HPH probes, respectively. WT, Wild type. [file Image_1.TIF]

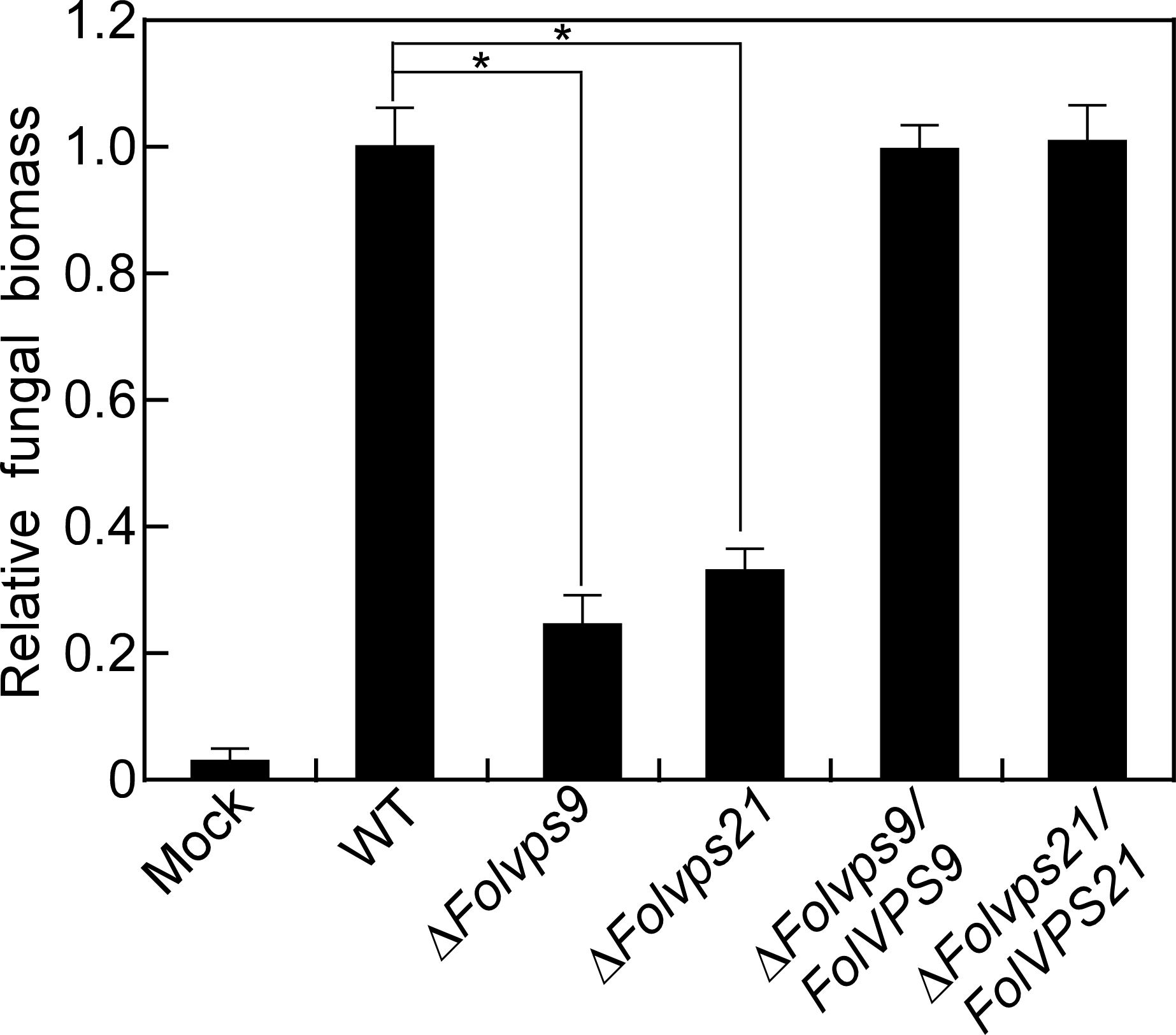

Supplement: FIGURE S2 — qPCR to determine relative levels of Fol in stem sections of inoculated tomato plants. Genomic DNA was isolated from tomato stems of the Moneymaker cultivar infected with various Fol strains as described in section Materials and Methods. Quantitative PCRs were performed to evaluate the fungal biomass using primers that amplify the intergenic spacer region of the ribosomal 28S subunit. The mean values of three determinations with standard deviations are shown. Asterisks indicate statistically significant difference relative to wild type Fol (p < 0.01). [file Image_2.TIF]

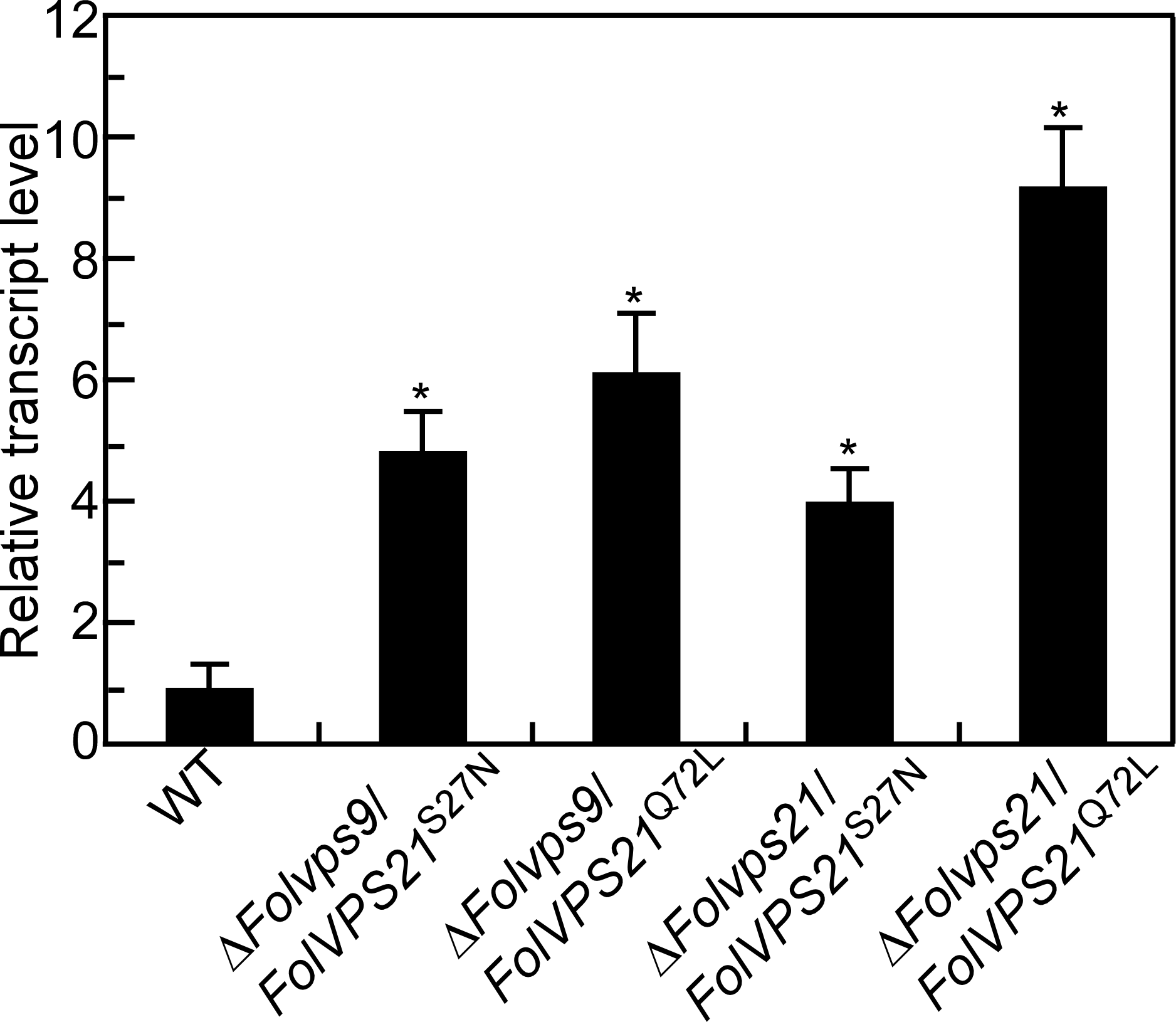

Supplement: FIGURE S3 — Expression of FolVPS21 in the indicated strains. The expression of FolVPS21 was measured by quantitative real-time PCR with cDNA isolated from hyphae of the indicated strains. Levels were first normalized to actin. The relative abundance of FolVPS21 transcripts was then normalized to that in wild-type vegetative hyphae (Relative transcript level = 1). Error bars represent ± SD and asterisks indicate statistically significant differences (p < 0.01). [file Image_3.TIF]
