# Supplementary material for: FolVps9, a Guanine Nucleotide Exchange Factor for FolVps21, Is Essential for Fungal Development and Pathogenicity in Fusarium oxysporum f. sp. lycopersici
Source: Front Microbiol. 2019 Nov 14;10:2658. doi: 10.3389/fmicb.2019.02658 (PMC6868059; doi:10.3389/fmicb.2019.02658)
Supplement: TABLE S1 — Wild-type and mutant strains of fungi used in this study. [file Data_Sheet_1.docx]

Table S1. Wild-type and mutant strains of fungi used in this study

| **Strain** | **Genotype description** | **Reference** |
| --- | --- | --- |
| *Fol4287* | Wild-type | Ouyang *et al*., (2014) |
| Δ*Folvps9* | *FolVPS9* deletion mutant of *Fol4287* | This study |
| Δ*Folvps9*/*FolVPS9* | Δ*Folvps9* mutant strain expressing GFP-FolVps9 construct | This study |
| Δ*Folvps9*/*FolVPS9*^ΔVPS9^ | Δ*Folvps9* mutant strain expressing GFP-FolVps9^ΔVPS9^ construct | This study |
| Δ*Folvps9*/*FolVPS9*^ΔCUE^ | Δ*Folvps9* mutant strain expressing GFP-FolVps9^ΔVPS9^ construct | This study |
| WT*/*GFP-FolAtg8 | Wild-type strain expressing GFP-FolAtg8 construct | This study |
| Δ*Folvps9/* GFP-FolAtg8 | Δ*Folvps9* mutant expressing GFP-FolAtg8 construct | This study |
| GFP*-*FolVps9*+*RFP-FolVps21 | Wild-type strain expressing GFP-FolVps9 and RFP-FolVps21 constructs | This study |
| FolVps9-CYFP+FolVps21^S27N^-NYFP | Wild-type strain expressing FolVps9-CYFP and FolVps21^S27N^-NYFP constructs | This study |
| FolVps9-CYFP+ NYFP | Wild-type strain expressing FolVps9-CYFP and NYFP constructs | This study |
| FolVps21-NYFP+ CYFP | Wild-type strain expressing FolVps21-NYFP and CYFP constructs | This study |
| CYFP+ NYFP | Wild-type strain expressing NYFP and CYFP constructs | This study |
| FolVps9-Flag+GFP-FolVps21^S27N^ | Wild-type strain expressing FolVps9-Flag and GFP-FolVps21^S27N^ constructs | This study |
| Δ*Folvps21* | *FolVPS21* deletion mutant of *Fol4287* | This study |
| Δ*Folvps21*/*FolVPS21* | Δ*Folvps21* mutant strain expressing RFP-FolVps21 construct | This study |
| Δ*Folvps21*/*FolVPS21*^S27N^ | Δ*Folvps21* mutant strain expressing RFP-FolVps21^S27N^ construct | This study |
| Δ*Folvps21*/*FolVPS21*^Q72L^ | Δ*Folvps21* mutant strain expressing RFP-FolVps21^Q72L^ construct | This study |
| Δ*Folvps9*/*FolVPS21*^S27N^ | Δ*Folvps9* mutant strain expressing RFP-FolVps21^S27N^ construct | This study |
| Δ*Folvps9*/*FolVPS21*^Q72L^ | Δ*Folvps9* mutant strain expressing RFP-FolVps21^Q72L^ construct | This study |
| Δ*Folvps9*+*FolVPS21*^S27N^+GFP-FolAtg8 | Δ*Folvps9* mutant strain expressing RFP-FolVps21^S27N^ and GFP-FolAtg8 construct | This study |
| Δ*Folvps9*+*FolVPS21*^Q72L^+GFP-FolAtg8 | Δ*Folvps9* mutant strain expressing RFP-FolVps21^Q72L^ and GFP-FolAtg8 construct | This study |
