# Supplementary material for: FolVps9, a Guanine Nucleotide Exchange Factor for FolVps21, Is Essential for Fungal Development and Pathogenicity in Fusarium oxysporum f. sp. lycopersici
Source: Front Microbiol. 2019 Nov 14;10:2658. doi: 10.3389/fmicb.2019.02658 (PMC6868059; doi:10.3389/fmicb.2019.02658)
Supplement: TABLE S2 — Primers used in this study. [file Data_Sheet_2.docx]

Table S2. Primers used in this study.

| Primer | Sequence (5’-3’) | Application |
| --- | --- | --- |
| *FolVPS9*-1F | CGATGATGTCTGTTCAAGGATGGT | Amplify *FolVPS9* 5’ flank sequence, for gene knock out |
| *FolVPS9*-2R | TTGACCTCCACTAGCTCCAGCCAAGCCGTTCGACGGCGAGGTAGCTGTTT |  |
| *FolVPS9-3F* | CAAAGGAATAGAGTAGATGCCGACCGCAACTGGGGAGCAGATAATTGTTG | Amplify *FolVPS9* 3’ flank sequence, for gene knock out |
| *FolVPS9-4R* | TGAATAGTCTGAACCCGGATATCC |  |
| *FolVPS9-5F* | AGGCCAACACCAAGAGGATGTTGA | Amplify *FolVPS9* gene probe, for southern blot and transformants screen |
| *FolVPS9-6R* | ATGAACTGAACAGCACCCATCTAC |  |
| *FolVPS9*-7F | TGTTGGCATTATGGAGAAGTCGA | Transformants screen |
| *FolVPS9*-8R | TAGCTTATCTGATGGAATGCCAT |  |
| HYG/F | GGCTTGGCTGGAGCTAGTGGAGGTCAA | Amplify *HPH-N* sequence |
| HY/R | GTATTGACCGATTCCTTGCGGTCCGAA |  |
| YG/F | GATGTAGGAGGGCGTGGATATGTCCT | Amplify *HPH-C* sequence |
| HYG/R | CGGTCGGCATCTACTCTATTCCTTTG |  |
| *FolVPS21*-1F | CGTCGAGATCTTCAGTAGCAAGAA | Amplify *FolVPS21* 5’ flank sequence, for gene knock out |
| *FolVPS21*-2R | TTGACCTCCACTAGCTCCAGCCAAGCCGGAGATGAGGTCAAGTTGGATGA |  |
| *FolVPS21-3F* | CAAAGGAATAGAGTAGATGCCGACCGAGTCAAGTGGCGTATGGGTCGAT | Amplify *FolVPS21* 3’ flank sequence, for gene knock out |
| *FolVPS21-4R* | TGGTGCAACAGAATCACAGTCACT |  |
| *FolVPS21-5F* | CTCCTAGCATACTAATTGAGCTG | Amplify *FolVPS21* gene probe, for southern blot and transformants screen |
| *FolVPS21-6R* | TCGGTAACGTTATGTCCGCTCTT |  |
| *FolVPS21*-7F | GGGCTTGTAGAAGGTCACTTCCTT | Transformants screen |
| *FolVPS21*-8R | AGGGCAGAGCCTAAGATAGCAGAA |  |
| GFP-FolVps9-1F | TTTCGTAGGAACCCAATCTTCAAAATGGTGAGCAAGGGCGAGGAGCTG-3’  GFP | *For GFP-FolVPS9* fusion construct, constitutive promoter |
| GFP- FolVps9-2R | CTTGTACAGCTCGTCCATGCCGAG |  |
| GFP- FolVps9-3F | CTCGGCATGGACGAGCTGTACAAGATGTCTCCGCCGGAGCAATCCAC |  |
| GFP- FolVps9-4R | CACCACCCCGGTGAACAGCTCCTCGCCCTTGCTCACTTATGTTGAAAGAGCGAGACACG |  |
| GFP-1F | TTTCGTAGGAACCCAATCTTCAAAATGGTGAGCAAGGGCGAGGAG | *For GFP-FolVPS9*^ΔVPS9^ fusion construct, constitutive promoter |
| GFP-2R | CTTGTACAGCTCGTCCATGCCGAG |  |
| FolVps9^ΔVPS9^-1F | CTCGGCATGGACGAGCTGTACAAGATGTCTCCGCCGGAGCAATCCAC |  |
| FolVps9^ΔVPS9^ -2R | CCGACGTCCACTGTCGCCGACTG |  |
| FolVps9^ΔVPS9^-3F | CAGTCGGCGACAGTGGACGTCGGGTGGAAGAGGCCGTCTCAGCAAT |  |
| FolVps9^ΔVPS9^-4R | CACCACCCCGGTGAACAGCTCCTCGCCCTTGCTCACTTATGTTGAAAGAGCGAGACACG |  |
| GFP-1F | TTTCGTAGGAACCCAATCTTCAAAATGGTGAGCAAGGGCGAGGAGCTG | *For GFP-FolVPS9*^ΔCUE^ fusion construct, constitutive promoter |
| GFP-2R | CTTGTACAGCTCGTCCATGCCGAG |  |
| FolVps9^ΔVPS9^ -1F | CTCGGCATGGACGAGCTGTACAAGATGTCTCCGCCGGAGCAATCCAC |  |
| FolVps9^ΔVPS9^ -2R | ACCCCGGTGAACAGCTCCTCGCCCTTGCTCACTTATGTTGCACGGGAAAGTCGCTGAG |  |
| RFP-FolVps21-1F: | TTTCGTAGGAACCCAATCTTCAAAATGGCCTCCTCCGAGGACGTCATC | *For RFP-FolVPS21* fusion construct, native promoter |
| RFP-FolVps21-2R: | GGGGCGTTGGCAGAGTCGGCCATGGCGCCGGTGGAGTGGCGGCCCT |  |
| RFP-FolVps21-3F: | ATGGCCGACTCTGCCAACGCCCC |  |
| RFP-FolVps21-4R: | CACCACCCCGGTGAACAGCTCCTCGCCCTTGCTCACCTAGCAAGCACAGCTGTCCTTAG |  |
| GFP-1F | TTTCGTAGGAACCCAATCTTCAAAATGGTGAGCAAGGGCGAGGAGCTG | *For GFP-FolATG8* fusion construct |
| GFP-2R | CTTGTACAGCTCGTCCATGCCGAG |  |
| GFP-FolAtg8-1F | CTCGGCATGGACGAGCTGTACAAGATGCGCAGCAAGTTCAAGGACGAG |  |
| GFP-FolAtg8-2R | CACCACCCCGGTGAACAGCTCCTCGCCCTTGCTCACTTACTCCCAAGCATCACCGAAGG |  |
| FolVps9-Flag-1F | CTATAGGGCGAATTGGGTACTCAAATTGGTTATGAAGTGCTCGTGTTTCATT | *For FolVPS9-Flag* fusion construct, native promoter |
| FolVps9-Flag-2R | CTTTATAATCACCGTCATGGTCTTTGT AGTCTGTTGAAAGAGCGAGACACGCAT |  |
| BD-FolVps9-1F | AAGCTGATCTCAGAGGAGGACCTGATGTCTCCGCCGGAGCAATCCAC | *FolVps9* yeast expression construct, for yeast two hybrid |
| BD-FolVps9-2R | TTATGTTGAAAGAGCGAGACACGCATCAACAGCCAGGCCGACCCTGCCCTCCTTCTCGT |  |
| AD-FolVps21-1F | TAACATATGATGGCCGACTCTGCCAACGCCCC | *FolVps21* yeast expression construct, for yeast two hybrid |
| AD-FolVps21-2R | TAAGAATTCCTAGCAAGCACAGCTGTCCTTA |  |
| BD-FolVps9^ΔVPS9^-1F | AAGCTGATCTCAGAGGAGGACCTGATGTCTCCGCCGGAGCAATCCAC | FoVps9^ΔVPS9^ yeast expression construct, for yeast two hybrid |
| BD- FoVps9^ΔVPS9^-2R | CCGACGTCCACTGTCGCCGACT |  |
| BD- FoVps9^ΔVPS9^-3F | AGTCGGCGACAGTGGACGTCGGGTGGAAGAGGCCGTCTCAGCAA |  |
| BD-FolVps9-2R | TTATGTTGAAAGAGCGAGACACGCATCAACAGCCAGGCCGACCCTGCCCTCCTTCTCGT |  |
| BD-FolVps9^ΔGUE^-1F | AAGCTGATCTCAGAGGAGGACCTGATGTCTCCGCCGGAGCAATCCAC | FolVps9^ΔGUE^ yeast expression construct, for yeast two hybrid |
| BD- FoVps9^ΔGUE^-2R | TGCAGGTCGACGGATCCCCGGTTATGTTGCACGGGAAAGTCGCTGAGCC |  |
| AD-FolVps21^S27N^-1F | TAACATATGATGGCCGACTCTGCCAACGCCCC | FolVps21^S27N^  yeast expression construct, for yeast two hybrid |
| AD-FolVps21^S27N^-2R | AACGAGAGAGTTCTTTCCGACG |  |
| AD-FolVps21^S27N^-3F | CGTCGGAAAGAACTCTCTCGTT |  |
| AD-FolVps21^S27N^-4R | TAAGAATTCCTAGCAAGCACAGCTGTCCTTA |  |
| AD-FolVps21^Q72L^-1F | TAACATATGATGGCCGACTCTGCCAACGCCCC | FolVps21^Q72L^  yeast expression construct, for yeast two hybrid |
| AD-FolVps21^Q72L^-2R | AGTGAGGCGAAGCGCTCCAGGC |  |
| AD-FolVps21^Q72L^-3F | GCCTGGAGCGCTTCGCCTCACT |  |
| AD-FolVps21^Q72L^-4R | TAAGAATTCCTAGCAAGCACAGCTGTCCTTA |  |
| pHZ68-FolVps21-1F | CGACTCACTATAGGGCGAATTGGGTACTCAAATTGAACATCAACCTCGGTGATGGCCA | FolVps21 fusion expression construct, for BiFC |
| pHZ68-FolVps21-2R | GTTCGGGATCTTGCAGGCCGGGCGGCAAGCACAGCTGTCCTTAGCAC |  |
| pHZ65-FolVps9-1F | CGACTCACTATAGGGCGAATTGGGTACTCAAATTGGCGATGAAGTGCTCGTGTTTCAT | FolVps9 fusion expression construct, for BiFC |
| pHZ65-FolVps9-2R | GCTCACCATCGTGGCGATGGAGCGACATCCTCTTGGTGTTGGCCTCGC |  |
| GFP-1F | TTTCGTAGGAACCCAATCTTCAAAATGGTGAGCAAGGGCGAGGAGCTG | GFP-FolVps21^S27N^ fusion expression construct, for Co-IP |
| GFP-2R | CTTGTACAGCTCGTCCATGCCGAG |  |
| GFP-FolVps21^S27N^-1F | CTCGGCATGGACGAGCTGTACAAGATGATGGCCGACTCTGCCAACGCCCC |  |
| GFP-FolVps21^S27N^-2R | AACGAGAGAGTTCTTTCCGACG |  |
| GFP-FolVps21^S27N^-3F | CGTCGGAAAGAACTCTCTCGTT |  |
| GFP-FolVps21^S27N^-4R | CACCACCCCGGTGAACAGCTCCTCGCCCTTGCTCACGCAAGCACAGCTGTCCTTAGC |  |
| RFP-1F | TTTCGTAGGAACCCAATCTTCAAAATGGCCTCCTCCGAGGACGTCATC | RFP-FolVps21^S27N^ fusion expression construct, constitutive promoter |
| RFP-2R | GGGGCGTTGGCAGAGTCGGCCATGGCGCCGGTGGAGTGGCGGCCCT |  |
| RFP-FolVps21^S27N^-1F | ATGGCCGACTCTGCCAACGCCCC |  |
| RFP-FolVps21^S27N^-2R | AACGAGAGAGTTCTTTCCGACG |  |
| RFP-FolVps21^S27N^-3F | CGTCGGAAAGAACTCTCTCGTT |  |
| RFP-FolVps21^S27N^-4R | CACCACCCCGGTGAACAGCTCCTCGCCCTTGCTCACCTAGCAAGCACAGCTGTCCTTAG |  |
| RFP-1F | TTTCGTAGGAACCCAATCTTCAAAATGGCCTCCTCCGAGGACGTCATC | RFP-FolVps21^Q72L^ fusion expression construct, constitutive promoter |
| RFP-2R | GGGGCGTTGGCAGAGTCGGCCATGGCGCCGGTGGAGTGGCGGCCCT |  |
| RFP-FolVps21^Q72L^-1F | ATGGCCGACTCTGCCAACGCCCC |  |
| RFP-FolVps21^Q72L^-2R | AGTGAGGCGAAGCGCTCCAGGC |  |
| RFP-FolVps21^Q72L^-3F | GCCTGGAGCGCTTCGCCTCACT |  |
| RFP-FolVps21^Q72L^-4R | CACCACCCCGGTGAACAGCTCCTCGCCCTTGCTCACCTAGCAAGCACAGCTGTCCTTAG |  |
| FolVps21-qRT-1F | CTCTCGTCGTTTATGATCTCACC | For constitutive transformant qRT-PCR analysis |
| FolVps21-qRT-2R | TCTTCTGTTGGAACCTTGCGTG |  |
| IGS-1F | TGCGATTTGGACGAGATATGTG | *For* determination *Fol* biomass |
| IGS-2R | ATTTGCCTACCCTGTACCTACC |  |
| SlActin-1F | CTCTCAAGTACCCTATTGAGCAT | Internal for analysis Fol biomass |
| SlActin-2R | CAATACCGGTAGTACGACCACT |  |
